# Vascular Endothelial Function as a Valid Predictor of Variations in Pulmonary Function in T2DM Patients Without Related Complications

**DOI:** 10.3389/fendo.2021.622768

**Published:** 2021-03-11

**Authors:** He Tai, Xiao-lin Jiang, Si-cheng Yao, Ye Liu, Hong Wei, Ling-bing Li, Zeng-jin Jiao, Tian-qing Wang, Jin-song Kuang, Lian-qun Jia

**Affiliations:** ^1^ Key Laboratory of Ministry of Education for Traditional Chinese Medicine Visera-State Theory and Application, Liaoning University of Traditional Chinese Medicine, Shenyang, China; ^2^ Department of Endocrinology and Metabolic, Liaoning Provincial Corps Hospital of Chinese People’s Armed Police Forces, Shenyang, China; ^3^ Department of Medical Laboratory, The Fourth of Affiliated Hospital of Guangzhou University of Traditional Chinese Medicine (Shenzhen Traditional Chinese Medicine Hospital), Guangzhou University of Traditional Chinese Medicine, Dalian, China; ^4^ Department of Echocardiography, The First Affiliated Hospital of Dalian Medical University, Beijing, China; ^5^ Department of Graduate School, China PLA General Hospital, Shenyang, China; ^6^ Department of Geriatrics, The Affiliated Hospital of Liaoning Traditional Chinese Medicine, Shenyang, China; ^7^ Department of Clinical Nutrition, The Second Affiliated Hospital of Liaoning University of Traditional Chinese Medicine, Shenyang, China; ^8^ Department of Endocrinology and Metabolic, Shenyang the Fourth Hospital of People, Shenyang, China

**Keywords:** pulmonary function, type 2 diabetes mellitus, vascular endothelial function, early stage, predictor

## Abstract

To assess the variations in pulmonary function and vascular endothelial function in their early stages (without related complications). A total of 162 type 2 diabetes mellitus (T2DM) patients without diabetes complications and 55 healthy people were selected, comprising the T2DM group and the control group, respectively, to evaluate changes in vascular endothelial function and lung function and determine the correlation between them. In this study, the T2DM group exhibited significantly lower pulmonary function than that of the control group (*P* < 0.05). The T2DM group also showed significantly lower flow-mediated dilation (FMD) and nitric oxide (NO) (*P* < 0.05) than those of the control group. Pulmonary functional indexes correlated positively with FMD and NO (*P* < 0.05) and correlated negatively with endothelin-1 (ET-1) (*P* < 0.05). FMD and NO correlated negatively with diabetes duration/HbA1c (*P* < 0.05), whereas ET-1 correlated positively with glycosylated hemoglobinA1c (HbA1c)/diabetes duration (*P* < 0.05). Pulmonary functional indexes negatively correlated with HbA1c/diabetes duration (*P* < 0.05). Multiple linear regression was used to analyze the relationship between vascular endothelial function indexes (FMD, ET-1, and NO) and pulmonary functional indexes. The results indicated that each vascular endothelial function index (FMD, ET-1, and NO) was significantly correlated with the pulmonary functional index (*P* < 0.05). The patients with T2DM presented changes in the subclinical vascular endothelial and pulmonary function. They also had impaired vascular endothelial functions, which were characterized by reduced vascular endothelial function relative to those of healthy people. Regulating glycemia may improve vascular endothelial and pulmonary functions. Moreover, microvascular lesions in preclinical stages, vascular endothelial function indexes (FMD, ET-1, and NO) were valid predictors of alterations in pulmonary function in T2DM patients without related complications.

**Clinical Trial Registration:**

ClinicalTrials.gov, identifier NCT03575988.

## Introduction

Type 2 diabetes mellitus (T2DM) is one of the most common chronic diseases worldwide. As a metabolic disorder (characterized by a relative lack of insulin), the prevalence of T2DM has increased worldwide, particularly in developing countries (1). The International Diabetes Federation estimates that by 2045, the prevalence of diabetes mellitus will increase to 693 million globally (2). Disabilities related to diabetes mellitus have grown substantially over the past decades. Specifically, T2DM markedly increases the risk for acute and chronic atherosclerotic cardiovascular disease (CVD) despite adequate glycemic control. T2DM is an independent risk factor for microvascular lesions (such as diabetes retinopathy [DR] and diabetic nephropathy [DN]) and cardiovascular disease (3, 4).

An early subclinical (without related complications) vascular effect in the preclinical stages of T2DM was represented by the endothelial dysfunction of conductance and resistance arteries (5). This could be explained by the effects of hyperglycemia and insulin resistance, resulting in the reduced bioavailability of vascular nitric oxide (NO) and inappropriate production of oxygen-free radicals (6). These alterations have been reported to be detectable in prediabetes (an intermediate stage along the continuum from normal glucose levels to the clinical entity T2DM) (7). Moreover, evidence shows that endothelial dysfunction may not only be the consequence of but also precede or even predict the onset of T2DM. Most studies only use the plasma biomarkers of endothelial activation or the FMD of the brachial artery in small study samples (8).

More than 30 years ago, the alveolar gas exchange capacity of patients with T2DM was found to be less than that of healthy people (9). However, although treatment of diabetic complications was attempted, diabetic lung injuries were overlooked (10). No studies have thus far been reported on the correlation between vascular endothelial function and pulmonary function in patients with T2DM. The correlation between vascular endothelial function indexes and the pulmonary functional indexes is significant for understanding the relationship that can lead to the prediction and prevention of diabetes complications and the changes in endothelial function and pulmonary functions in T2DM adults without related complications.

## Methods

### Subjects

A total of 180 T2DM patients without related complications were recruited as the diabetes group, and 60 healthy subjects were recruited as the control group. All patients were from the Department of Endocrinology and Metabolism at the Fourth People’s Hospital of Shenyang. The clinical research protocol was approved by the medical ethics committee (number ICE2018052802) of the Fourth People’s Hospital of Shenyang.

All participants were from the Chinese ethnic group Han. Therefore, all recruited patients were advised to follow a diet and exercise program formulated by dieticians. No significant differences in sex ratio and age (range=36–67 y) were found between the groups (*P* > 0.05). Their diabetes durations (range=4–12 y), HbA1c (range=6.7%–9.8% [50.82 mmol/mol–83.61 mmol/mol]), indicated pulmonary functional indexes, indicated vascular endothelial function indexes, indicated blood lipid indexes, and blood pressure (BP) were determined (**Table 1**). T2DM and related complications were diagnosed in accordance with the *Guidelines of Diabetes* (11).

**Table 1:** Baseline demographic and clinical characteristics.

Characteristics		Control group	Diabetes group	*t*/χ^2^ value	*P* value
NO. (*n*)		55	162	–	–
Sex, *n* (%)	Male	30 (54.5)	86 (53.1)	0.035	0.851
	Female	25 (45.5)	76 (46.9)		
Age (years)		50.80 ± 7.07	51.99 ± 7.91	0.987	0.325
Diabetes duration (years)		–	8.03 ± 1.95	–	–
FBG (mmol/l)		4.99 ± 0.47	8.11 ± 0.47	42.279	<0.0001
2 hPBG (mmol/l)		7.31 ± 0.77	11.93 ± 0.69	41.642	<0.0001
HbA1c	%	5.04 ± 0.40	8.13 ± 0.74	29.418	<0.0001
	mmol/mol	31.56 ± 4.37	65.30 ± 8.11	29.418	<0.0001
Baseline HbA1c, *n* (%)	≤7%/(53 mmol/mol)	55 (100.0)	16 (9.88)	151.496	<0.0001
	>7%/(53 mmol/mol)	0 (0.0)	146 (90.12)		
TC (mg/dl)		186.82 ± 19.64	207.44 ± 18.81	6.949	<0.0001
HDL-C (mg/dl)		38.62 ± 3.58	46.49 ± 4.13	-13.528	<0.0001
LDL-C (mg/dl)		109.82 ± 7.54	112.66 ± 11.26	1.742	0.083
TG (mg/dl)		123.02 ± 11.23	163.90 ± 18.69	15.301	<0.0001
SBP (mmHg)		124.47 ± 6.93	129.65 ± 4.82	6.107	<0.0001
DBP (mmHg)		87.42 ± 6.10	91.45 ± 5.38	4.639	<0.0001
NO (µmol/L)		91.31 ± 2.06	76.39 ± 6.37	-17.047	<0.0001
ET-1 (pg/ml)		74.38 ± 4.27	147.15 ± 10.26	51.071	<0.0001
FMD (%)		8.70 ± 0.30	6.78 ± 0.13	-64.938	<0.0001
VC Litre (% of predicted)		87.03 ± 3.20	80.99 ± 4.15	-9.843	<0.0001
FVC Litre (% of predicted)		80.58 ± 2.11	74.26 ± 2.34	-17.699	<0.0001
FEV1 Litre (% of predicted)		78.22 ± 2.14	74.64 ± 2.13	-10.747	<0.0001
PEF L/S (% of predicted)		59.01 ± 2.35	50.46 ± 2.89	-19.853	<0.0001
MVV Litre (% of predicted)		88.84 ± 1.97	82.73 ± 3.71	-11.657	<0.0001
TLC Litre (% of predicted)		95.91 ± 1.57	91.95 ± 2.39	-11.466	<0.0001
FEV1/FVC (% of predicted)		79.64 ± 2.69	74.55 ± 2.40	-13.151	<0.0001
DLCO (mL/min/mmHg) (% of predicted)		87.67 ± 3.01	81.25 ± 4.18	-10.500	0.001
DLCO/VA(mL/min/mmHg) (% of predicted)		88.29 ± 2.80	82.65 ± 7.20	-5.663	<0.0001

FBG, fasting plasma glucose; 2hPBG, 2-hour postprandial blood glucose; HbA1c, glycosylated hemoglobinA1c; TC, Total cholesterol; HDL-C, High-density lipoprotein cholesterol; LDL-C, Low-density lipoprotein cholesterol; TG, Triglycerides; SBP, systolic blood pressure; DBP, diastolic blood pressure; FMD, flow-mediated dilation; NO, nitric oxide; ET-1, endothelin-1; VC, vital capacity; FVC, forced vital capacity; FEV1, forced expiratory volume in 1 second; PEF, peak expiratory force; MVV, maximal voluntary ventilation; TLC, total lung capacity; FEV1/FVC, forced expiratory volume in 1 second/forced vital capacity; DLCO, diffusing capacity for carbon monoxide of lung; DLCO/VA, diffusing capacity for carbon monoxide of lung/unit volume.

The following patients were included in the study: 1) patients with T2DM were recruited as defined by the *Guidelines of Diabetes* (11); 2) patients with no history of pulmonary disease, smoking, and recent viral-related diseases; 3) patients without hepatopathy, nephropathy, hyperuricemia, or gastrointestinal disease; and 4) patients likely to comply with the study guidelines who could visit the hospital for periodic assessments. The following were excluded from this study: 1) patients with type 1 diabetes mellitus, patients with gestational diabetes, and lactating patients; 2) patients with microvascular lesions (such as DN and DR); 3) patients whose liver enzyme levels were two times higher than normal; 4) patients with inadequate renal function (induced by DN)—that is, patients with serum creatinine >132 µmol/L for males or 123 µmol/L for females or an AER >30 mg/24 h or 20 mg/min; 5) patients whose liver enzyme levels were two times higher than normal; 6) patients requiring intensive care and continuous insulin treatment; 7) patients with associated hypertension or patients receiving antihypertensive drugs; 8) patients who, according to the New York Heart Association, have heart failure (type III or IV), a coronary stent placement, history of coronary angioplasty, coronary bypass surgery, or even myocardial infarction (< 6 mo before study recruitment); 9) patients whose blood lipid levels were inadequately controlled by cholesterol-lowering drugs (i.e., patients with a TC >250 mg/dL, HDL-C <30 mg/dL, LDL-C >170 mg/dL, or TGs >200 mg/dL); and 10) patients treated with systemic injections of glucocorticoids within 3 months before enrollment in the study.

### Study Design

**Figure 1:**
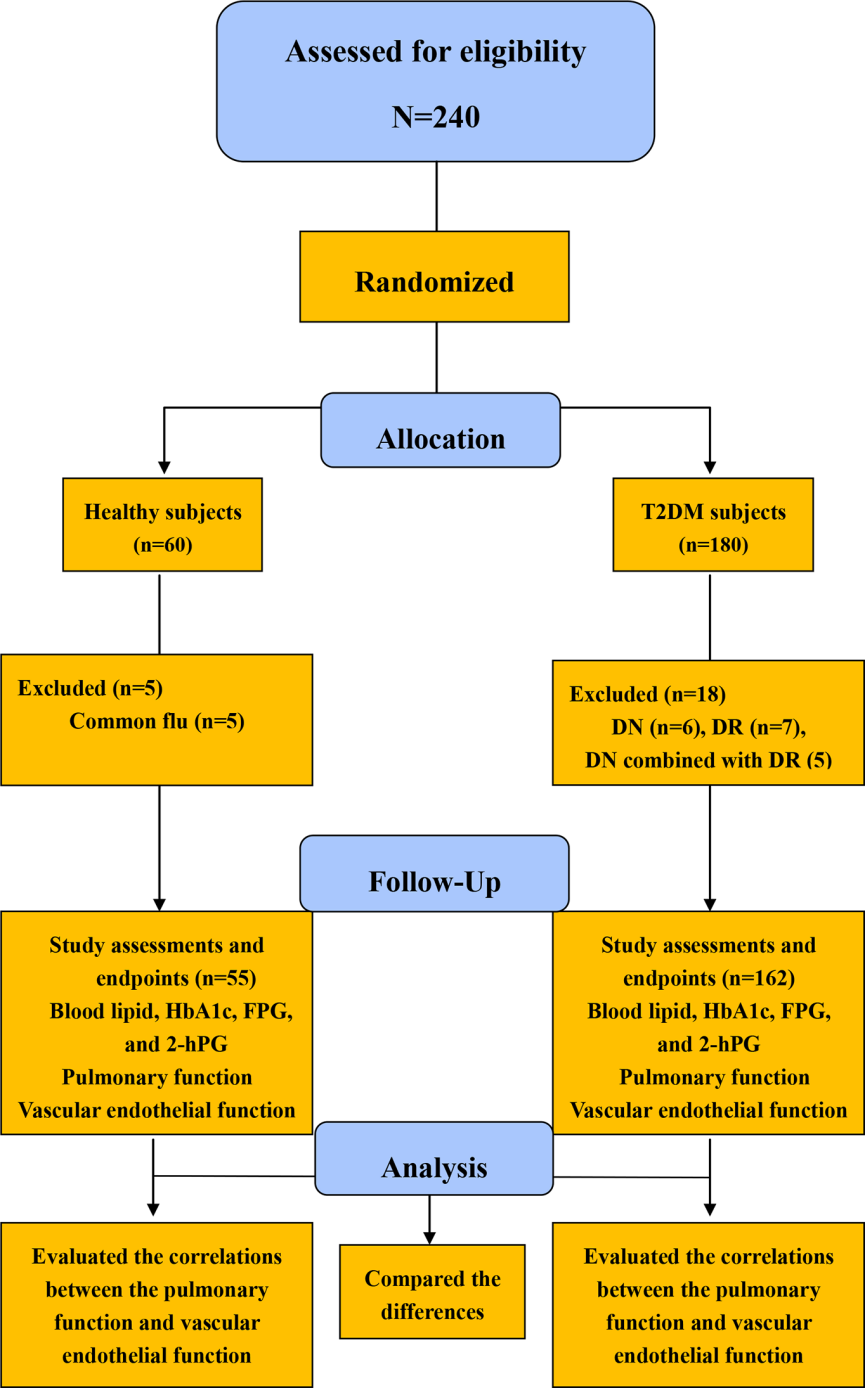
Flow diagram for the study sample in the present study HbA1c, glycosylated hemoglobinA1c; FBG, fasting plasma glucose; 2hPBG, 2-hour postprandial blood glucose.

A total of 180 patients with T2DM (95 males and 85 females) and 60 healthy subjects (32 males and 28 females), all of whom performed the indicated analysis tests to exclude DN and DR, were recruited in a single time-point measurement study phase. Following the study, 18 patients were excluded because of DN or DR combinations. Moreover, 5 healthy subjects were excluded because they contracted the common flu. Thus, 162 patients with T2DM and 55 healthy subjects were eligible to join in the subsequent study. The subjects fasted for at least 8 h to undergo the related laboratory test with 5 mL of venous blood. Tests for pulmonary function, intraocular pressure, and vascular endothelial function were also conducted. FMD was conducted *via* a color Doppler ultrasound (CDU). Ultimately, 162 patients (86 males and 76 females) and 55 healthy subjects (30 males and 25 females) completed this study (**Figure 1**).

### Study Assessments and Endpoints

Blood specimen collection and testing: Venous blood was collected between 0600 H and 0800 H (the patients fasted for at least 8 h), and the blood lipid, HbA1c, and FPG levels were determined. The 2 hPG levels were measured based on venous blood samples in accordance with the standards established in this study.

BP measurements: SBP and DBP were measured using an electronic sphygmomanometer.

Pulmonary function measurements: The indicated pulmonary functional indexes were measured with a spirometer. The effects of weight, height, and age were excluded using the measured-to-expected ratios and predicted value percentages.

Vascular endothelial function measurements: Endothelial function measurements were conducted in dark, air-conditioned rooms (23°C–25°C) after at least 5 min of rest and 8 h of fasting in the supine position. The subjects were particularly advised to refrain from nicotine, caffeine, alcohol, vitamins, and physical activity before the measurement. The artery diameter was gauged in resting conditions, and FMD was determined after a 5 min upper-arm occlusion under standardized conditions as a percentage increase in baseline artery diameter. Two-dimensional high-resolution ultrasound images of the right brachial artery were acquired using an ultrasound machine (Philips HD11XE CV) combined with a linear array broadband probe (38 mm L12-5). The software Brachial Analyzer version 5.0 (Medical Imaging Applications LLC, Iowa City, IA) was used to analyze the artery diameters, FMN= (line of maximum expansion-line of peacetime)/line of peacetime×100% (12). Plasma ET-1 and 10 mL of venous blood were collected in an ethylenediaminetetraacetic acid (EDTA) tube and then immediately centrifuged for 20 min at 4°C at 2500 g. Enzyme-linked immunosorbent assay kits (Morinaga and R and D System) measured the ET-1 (13). Reagents and test samples were prepared and assayed as specified in the instructions provided by the manufacturer. The plasma NO concentration was determined using the Griess method (14).

### Statistical Analysis

All calculations were performed using SPSS version 17.0 (SPSS Inc., Chicago, IL, USA). Continuous variables with normal distribution are presented as mean ± standard deviation (SD) to express the measurement data. Percentages were used to express the numerical data. Data were statistically analyzed using SPSS. Pearson’s correlation coefficient was utilized to evaluate the linear correlations between the pulmonary function and HbA1c/diabetes duration, vascular endothelial function indexes, and HbA1c/diabetes duration, pulmonary function, and vascular endothelial function indexes. Pulmonary functional indexes were dependent variables, the independent variables were independent variables, and the dependent and independent variables were used in multiple linear regression analysis. *P* < 0.05 was considered statistically significant.

## Results

### Participant Criterion Characteristics

The diabetes group consisted of 162 T2DM subjects (86 males and 76 females) with a mean diabetes duration of 8.03 ± 1.95 y, whereas the control group consisted of 55 subjects (30 males and 25 females) (**Figure 1**).

The pulmonary functional indexes and the vascular endothelial function indexes in the diabetes group were significantly greater than those in the control group (*P* < 0.05). Among the pulmonary functional indexes were triglycerides (TG), total cholesterol (TC), low-density lipoprotein cholesterol (LDL-C), glycosylated hemoglobinA1c (HbA1c), 2-hour postprandial blood glucose (2 hPG), and fasting plasma glucose (FPG). The high-density lipoprotein cholesterol (HDL-C) in the diabetes group was lower than that in the control group (*P* < 0.05); however, no significant differences in diastolic blood pressure (DBP) and systolic blood pressure were found between the two groups (*P* > 0.05) (**Table 1**).

### Correlation Between Pulmonary Functional Indexes and Diabetes Duration/Hba1c

A significant negative correlation was determined between pulmonary functional indexes and diabetes duration/HbA1c (*P* < 0.05) (**Table 2**); by contrast, such a negative correlation was not found to be significant in the control group (*P* > 0.05).

**Table 2:** Correlation of pulmonary function parameters to HbA1c and diabetes duration in diabetes group.

Pulmonary function parameters	HbA1c	Diabetes duration
VC	*r*=-0.902*	*r*=-0.350*
*P* value	< 0.0001	< 0.0001
FVC	*r*=-0.772*	*r*=-0.339*
*P* value	< 0.0001	< 0.0001
FEV1	*r*=-0.672*	*r*=-0.281*
*P* value	< 0.0001	< 0.0001
PEF	*r*=-0.844*	*r*=-0.306*
*P* value	< 0.0001	< 0.0001
MVV	*r*=-0.755*	*r*=-0.356*
*P* value	< 0.0001	< 0.0001
TLC	*r*=-0.838*	*r*=-0.339*
*P* value	< 0.0001	< 0.0001
FEV1/FVC	*r*=-0.731*	*r*=-0.296*
*P* value	< 0.0001	< 0.0001
DLCO	*r*=-0.879*	*r*=-0.334*
*P* value	< 0.0001	< 0.0001
DLCO/VA	*r*=-0.348*	*r*=-0.289*
*P* value	< 0.0001	< 0.0001

*P< 0.05, Statistically significant.

HbA1c, glycosylated hemoglobinA1c; VC, vital capacity; FVC, forced vital capacity; FEV1, forced expiratory volume in 1 second; PEF, peak expiratory force; MVV, maximal voluntary ventilation; TLC, total lung capacity; FEV1/FVC, forced expiratory volume in 1 second/forced vital capacity; DLCO, diffusing capacity for carbon monoxide of lung; DLCO/VA, diffusing capacity for carbon monoxide of lung/unit volume.

### Correlation Between Vascular Endothelial Function Indexes and Diabetes Duration/Hba1c

FMD and NO were found to exhibit a significant negative correlation with diabetes duration/HbA1c (*P* < 0.05) and endothelin-1 (ET-1) and a significant positive correlation with diabetes duration/HbA1c (*P* < 0.05) (**Table 3**); by contrast, such correlations exhibited no significance in the control group (*P* > 0.05).

**Table 3:** Correlation of vascular endothelial function parameters to HbA1c and diabetes duration in diabetes group.

Renal parameters	HbA1c	Diabetes duration
NO	*r*=-0.883*	*r*=-0.317*
*P* value	< 0.0001	< 0.0001
ET-1	*r*=0.905*	*r*= 0.398*
*P* value	< 0.0001	< 0.0001
FMD	*r*= -0.826*	*r*= -0.358*
*P* value	< 0.0001	< 0.0001

*P< 0.05, Statistically significant.

FMD, flow-mediated dilation; NO, nitric oxide; ET-1, endothelin-1; HbA1c, glycosylated hemoglobinA1c.

### Correlation Between Pulmonary Functional Indexes and Vascular Endothelial Function Indexes

A significant negative correlation was determined between pulmonary functional indexes and ET-1 (*P* < 0.05), whereas a significant positive correlation was found between pulmonary functional indexes and FMD/NO (*P* < 0.05) (**Table 4**); however, in the control group, such correlations showed no statistical significance (*P* > 0.05). Multiple linear regression analysis indicated that the 9 pulmonary functional indexes were significantly correlated with FMD (*P* < 0.05) (**Table 5**).

**Table 4:** Correlation between pulmonary function parameters and vascular endothelial function parameters in diabetes group.

Pulmonary function	Vascular endothelial function parameters		
	NO	ET-1	FMD
VC	*r*= 0.830*	*r*= -0.798*	*r*= 0.698*
*P* value	< 0.0001	< 0.0001	< 0.0001
FVC	*r*= 0.707*	*r*= -0.808*	*r*= 0.679*
*P* value	< 0.0001	< 0.0001	< 0.0001
FEV1	*r*= 0.643*	*r*= -0.717*	*r*= 0.606*
*P* value	< 0.0001	< 0.0001	< 0.0001
PEF	*r*= 0.775*	*r*= -0.797*	*r*= 0.678*
*P* value	< 0.0001	< 0.0001	< 0.0001
MVV	*r*= 0.682*	*r*= -0.798*	*r*= 0.722*
*P* value	< 0.0001	< 0.0001	< 0.0001
TLC	*r*= 0.739*	*r*= -0.770*	*r*= 0.567*
*P* value	< 0.0001	< 0.0001	< 0.0001
FEV1/FVC	*r*= 0.641*	*r*= -0.774*	*r*= 0.650*
*P* value	< 0.0001	< 0.0001	< 0.0001
DLCO	*r*= 0.797*	*r*= -0.787*	*r*= 0.700*
*P* value	< 0.0001	< 0.0001	< 0.0001
DLCO/VA	*r*= 0.318*	*r*= -0.376*	*r*= 0.309*
*P* value	< 0.0001	< 0.0001	< 0.0001

*P< 0.05, Statistically significant.

FMD, flow-mediated dilation; NO, nitric oxide; ET-1, endothelin-1; VC, vital capacity; FVC, forced vital capacity; FEV1, forced expiratory volume in 1 second; PEF, peak expiratory force; MVV, maximal voluntary ventilation; TLC, total lung capacity; FEV1/FVC, forced expiratory volume in 1 second/forced vital capacity; DLCO, diffusing capacity for carbon monoxide of lung; DLCO/VA, diffusing capacity for carbon monoxide of lung/unit volume; RI, resistivity index.

**Table 5:** Multiple regression analysis between pulmonary function and vascular endothelial function parameters in diabetes group.

Dependent variable	Coefficient					*P* value
	R	F	NO	ET-1	FMD	
VC	0.865	156.215	7.641	- 3.900	0.126	0.006*
FVC	0.822	109.365	1.992	- 6.467	2.282	<0.0001*
FEV1	0.730	60.213	2.024	- 4.699	1.248	<0.0001*
PEF	0.833	119.603	5.006	- 5.053	1.506	0.002*
MVV	0.818	106.415	1.177	- 6.065	3.507	0.001*
TLC	0.808	199.913	3.993	- 4.189	2.574	<0.0001*
FEV1/FVC	0.779	81.387	0.618	- 6.625	1.650	<0.0001*
DLCO	0.843	129.367	6.125	- 3.833	2.445	0.020*
DLCO/VA	0.379	398.745	3.618	- 2.181	0.370	0.080

*P< 0.05, Statistically significant.

FMD, flow-mediated dilation; NO, nitric oxide; ET-1, endothelin-1; VC, vital capacity; FVC, forced vital capacity; FEV1, forced expiratory volume in 1 second; PEF, peak expiratory force; MVV, maximal voluntary ventilation; TLC, total lung capacity; FEV1/FVC, forced expiratory volume in 1 second/forced vital capacity; DLCO, diffusing capacity for carbon monoxide of lung; DLCO/VA, diffusing capacity for carbon monoxide of lung/unit volume.

## Discussion

The concept that endothelial dysfunction may accelerate the progression of T2DM has been previously proposed. Hemostatic markers such as the von Willebrand factor and plasminogen activator inhibitor-1 and elevated endothelial dysfunction/activation biomarkers, including E-selectin, adhesion molecules, intercellular adhesion molecule 1, and vascular cell adhesion molecule 1, predicted an increased T2DM risk independent of other risk factors for diabetes mellitus. These factors include obesity, inflammation, and insulin resistance (8, 15). Rossi et al. (16) indicated that in postmenopausal women, endothelial dysfunction assessed *via* the brachial artery FMD might precede T2DM onset. This finding suggests that microvascular endothelial dysfunction can be detected before the development of (pre-) diabetes mellitus and precede the occurrence of (pre-) diabetes mellitus. This association was weaker for FMD, a measure of the endothelial function of large conductance vessels, allowing the differentiation of microvascular and macrovascular endothelial dysfunction for the development and progression of (pre-) diabetes mellitus. The mean FMD in the diabetes group (6.78% ± 0.13%) was significantly smaller than that in the control group (8.70% ± 0.30%) (*P* < 0.05). This result was consistent with previous studies (12, 16).

Pathophysiological endothelial dysfunction results from the following: recurrent hyperglycemia; elevated free fatty acids; systemic insulin resistance caused by impairment of insulin signaling; decreased vascular bioavailability of NO caused by reduced production and/or increased inactivation of NO; and increased oxidative stress with the consequence of enhanced vasoconstriction, inflammation, and thrombosis (17). Several methods for the *in vivo* assessment of endothelial dysfunction in humans can be achieved. For instance, using the FMD of the brachial artery to determine the endothelial function of arterioles is a valid approach to assessing the conduit artery and resistance artery endothelial dysfunction (18). The technique partly depends on NO and the correlates of coronary endothelial function. Notably, endothelial dysfunction in large and/or small arteries has been consistently linked to cardiovascular events in patients with arterial hypertension, peripheral artery disease, coronary artery disease, and heart failure (18, 19). The mean systolic blood pressure (SBP) and diastolic blood pressure (DBP) in the diabetes group (129.65 ± 4.82, 91.45 ± 5.38 mmHg) were significantly higher than those in the control group (124.47 ± 6.93, 87.42 ± 6.10 mmHg) (*P* < 0.05) (**Table 1**).

ET-1, which is generated by the vascular endothelium and released to sanguis, represents a potent endogenous vasoconstrictor peptide. Moreover, ET-1 has been associated with heart failure, hypertension, atherosclerotic vascular, and T2DM. As obesity increases, the induced ET-1 vasoconstrictor tone force increases, whereas the endothelium-dependent vasodilation decreases (20, 21). An original study found that culturing arterial endothelial cells and increasing their resistance could generate a significant reduction in endothelial cells (22). The results of the present study revealed that ET-1 in the diabetes group (147.15 ± 10.26 pg/mL) was significantly higher than that in the control group (74.38 ± 4.27 pg/mL) (*P* < 0.05) and that NO in the diabetes group (76.39 ± 6.37 µmol/L) was significantly lower than that in the control group (91.31 ± 2.06 µmol/L) (*P* < 0.05).

Therefore, we selected FMD, ET-1, and NO to assess the vascular endothelial function parameters.

As an indicator of glycemic control, HbA1c is widely used in clinical setting. A higher HbA1c level denotes poorer diabetes control and a higher serum glucose level. With a sustained high level (3 mo or longer) of circulating glucose, nonenzymatic glycosylation of tissue protein increases (23). In the present study, patients with low HbA1c (< 7.0% [53 mmol/mol]) were recruited; however, in the study by Prabhu M et al. (24), only a fraction (23 (11.5%)) of patients realized the significance of HbA1c and neglected blood sugar control. Moreover, in the current study, few patients achieved the targeted HbA1c level (7% [53 mmol/mol]), with a corresponding proportion of 9.88% (16/162). By contrast, most of the patients did not attain the target HbA1c control level (7% (53 mmol/mol)) and failed to lower their glycemia adequately. Therefore, the 2 hPG (11.93 mmol/L) and FPG (8.11 mmol/L) levels in the diabetes group were higher than the corresponding targets (10 mmol/L and 7 mmol/L).

The mechanism of lung injury caused by diabetes is ambiguous; however, glycemic control has the most important role in reducing lung injury in diabetes. Cavan DA et al. (25) found that the nonenzymatic glycosylation of lung proteins could reduce lung compliance, thereby inducing the lower alveolar–capillary microvascular reserve system and aggravating oxidative injury—that is, the hyperglycemic state damaged the lung and reduced lung function (26). A clinical study also found that hypoxia could induce chronic or acute pathological lung conditions (loss of microvascular reserve) (26).

The integrity of the pulmonary capillary endothelium can affect diffusing capacity of the lung for CO, suggesting the importance of pulmonary microvascular injury. Reports over the past 15 years on pulmonary function tested in diabetes patients have focused on pulmonary function. However, research focusing on the mechanism underlying pulmonary function injury is rarely reported. Lung functional indexes related to pulmonary microangiopathy include CO transfer capacity and pulmonary capillary blood volume (27). Niranjan V showed that the forced expiratory volume in 1 second (FEV1), vital capacity (VC), total lung capacity (TLC), and forced vital capacity (FVC) in type 1 diabetes patients were significantly lower than those in healthy people (28). However, the population of patients with T2DM in the present study was smaller than that in the report by Niranjan V (29). The results of the present study revealed that the pulmonary function indexes in the diabetes group were significantly lower than those in the control group. The pulmonary function indexes in the diabetes group were as follows: VC (80.99 ± 4.15), FVC (74.26 ± 2.34), FEV1 (74.64 ± 2.13), peak expiratory force (PEF) (50.46 ± 2.89), maximal voluntary ventilation (MVV) (82.73 ± 3.71), TLC (91.95 ± 2.39), FEV1/FVC (74.55 ± 2.40), DLCO (82.65 ± 7.20) (mL/min/mmHg), and DLCO/VA (82.65 ± 7.20) (mL/min/mmHg) (% of predicted). The pulmonary function indexes in the control group were as follows: VC (87.03 ± 3.20), FVC (80.58 ± 2.11), FEV1 (78.22 ± 2.14), PEF (59.01 ± 2.35), MVV (88.84 ± 1.97), TLC (95.91 ± 1.57), FEV1/FVC (79.64 ± 2.69), DLCO (87.67 ± 3.01), and DLCO/VA (88.29 ± 2.80) (mL/min/mmHg) (mL/min/mmHg) (% of predicted)) (*P* < 0.05). Lange P et al. (30) identified an inconsistency in the correlations between pulmonary function and HbA1c. Meanwhile, the present study demonstrated the weak correlations between pulmonary function indexes and HbA1c, as well as the strong correlations between pulmonary function indexes and diabetes duration. Another cross-sectional population research showed that serum glucose levels were negatively correlated with FVC and/or FEV (31). HbA1c was chosen as an indicator of diabetes control. In the present study, HbA1c was negatively correlated with pulmonary functional indexes (*P* < 0.05), but such correlations were not statistically significant in the control group (*P* > 0.05). In the diabetes group, diabetes duration exhibited statistically significant correlations with pulmonary functional indexes (*P* < 0.05), and pulmonary functional indexes were negatively correlated with HbA1c/diabetes duration (**Table 2**). These outcomes were consistent with the original studies (30, 31).

From a pathophysiological perspective, two mechanisms could explain the association between microvascular alterations and T2DM risk (32, 33). First, from the arteriolar microcirculation, endothelial dysfunction may damage insulin function to redirect blood flow in the skeletal muscle and reduce glucose uptake, which was mediated by insulin. Second, pancreatic microvascular endothelial dysfunction has a vital function. It can cause β cell apoptosis in the pancreas, reducing insulin secretion and then inducing hyperglycemia, further impairing the microvascular endothelial function (34, 35). Vascular endothelial function indexes showed a statistically significant correlation with HbA1c/diabetes duration in the diabetes group (*P* < 0.05). NO, FMD, and HbA1c/diabetes duration were negatively correlated, whereas ET-1 and HbA1c/diabetes duration were positively correlated (**Table 3**). The outcomes were consistent with those of the original research (12).

In the present study, the correlations between vascular endothelial function indexes and pulmonary functional indexes were statistically significant (*P* < 0.05); NO/FMD and pulmonary functional indexes were positively correlated, whereas ET-1 and pulmonary functional indexes were negatively correlated (**Table 4**). However, in the control group, these correlations were not statistically significant (*P* > 0.05). According to the present results, the vascular endothelial function indexes could be used to evaluate the interactions between them in the early stages of T2DM (without related complications). Moreover, multiple linear regressions showed statistically significant correlations between the pulmonary functional indexes and the vascular endothelial function indexes (*P* < 0.05). NO had the highest VC (7.641), DLCO (6.125), and DLCO/VA (3.618) coefficients; ET-1 had the highest FVC (- 6.467), FEV1 (- 4.699), PEF (- 5.053), MVV (- 6.065), TLC (- 4.189), and FEV1/FVC (- 6.625) coefficients (**Table 5**). Therefore, the results of this multiple linear regression analysis indicated that the vascular endothelial function indexes could be used as strong predictors of pulmonary function in T2DM adults without related complications.

This study indicated that in addition to the vascular endothelial function indexes, the consociation of the retrobulbar resistivity index (RI) (36) and HbA1c could be seen as good predictors of subclinical changes in diabetes patients. The association predictive significance was better than either of the indexes on their own.

However, the present study had several deficiencies, which should be addressed in future research. First, the change in alveolar tissue morphology induced by diabetes was not observed. The mechanism of the injury, which included specific proteins and genes, was not investigated because the patients could not receive lung biopsies; thus, animal models will be used to research the mechanism. Second, no longitudinal follow-up study was conducted; thus, long-term changes in the lung and endothelium must be observed in the future. The predictive value of the vascular endothelial function indexes in the early stages (without microangiopathy) should be evaluated. Moreover, patients with T2DM without complications were examined, but the predictive values in different T2DM phases were not evaluated. Therefore, clinicians should pay increased attention to the treatment and prevention of lung injury in patients with T2DM and discover more meaningful indicators to predict the degree of injury.

## Conclusions

Our results demonstrated that T2DM patients (without related complications) exhibited changes in subclinical vascular endothelial and pulmonary functions. The T2DM patients showed had impaired vascular endothelial functions, as characterized by reduced vascular endothelial function relative to that in healthy people. Regulating glycemia may improve vascular endothelial and pulmonary functions. In the preclinical stages of microvascular lesions, the vascular endothelial function indexes (FMD, ET-1, and NO) were found to be valid predictors of changes in pulmonary function in T2DM patients without related complications.

## Data Availability Statement

The data from the original study are available from Shenyang the Fourth Hospital of People *via* its ClinicalTrials.gov (NCT03575988). The datasets analyzed during the current study are available from all the authors on reasonable request.

## Ethics Statement

This clinical research protocol was approved by the medical ethics committee (number ICE2018052802) of the Fourth People’s Hospital of Shenyang. The patients/participants provided their written informed consent to participate in this study.

## Author Contributions

HT and X-lJ wrote the manuscript and analyzed the data. S-cY, YL, HW, and L-bL recruited the patients and collected the blood samples. Z-jJ generated the tables. YL, HW, and L-bL determined the related indexes. T-qW, J-sK, and L-qJ contributed to the discussion and reviewed the manuscript. All authors contributed to the article and approved the submitted version.

## Funding

Support was provided by the National Natural Science Foundation of China to L-qJ (81774022), Fund Project of Innovation Team in Liaoning to L-qJ (LT2016012), Inheritance and Innovation Project of Traditional Chinese Medicine (Qihuang Project), and “Xingliao Yingcai Project” of Liaoning Province.

## Conflict of Interest

The authors declare that the research was conducted in the absence of any commercial or financial relationships that could be construed as a potential conflict of interest.

## Abbreviations

FMD, flow-mediated dilation; T2DM, type 2 diabetes mellitus; CVD, cardiovascular disease; DN, diabetes nephropathy; DR, diabetes retinopathy; NO, nitric oxide; ET-1, endothelin-1; HbA1c, glycosylated hemoglobinA1c; FBG, fasting plasma glucose; 2hPBG, 2-hour postprandial blood glucose; RBS, random blood sugar; OGTT, oral glucose tolerance test; CDU, color Doppler ultrasound; EDTA, ethylenediaminetetraacetic acid; ELISA, enzyme linked immunosorbent assay; SD, standard deviation; VC, vital capacity; FVC, forced vital capacity; FEV1, forced expiratory volume in 1 second; PEF, peak expiratory force; MVV, maximal voluntary ventilation; TLC, total lung capacity; FEV1/FVC, forced expiratory volume in 1 second/forced vital capacity; DLCO, diffusing capacity for carbon monoxide of lung; DLCO/VA, diffusing capacity for carbon monoxide of lung/unit volume; CDU, color Doppler ultrasound; DR, diabetes retinopathy; RI, resistivity index; TC, total cholesterol; HDL-C, high-density lipoprotein cholesterol; LDL-C, low-density lipoprotein cholesterol; TG, triglycerides; SBP, systolic blood pressure; DBP, diastolic blood pressure; BP, blood pressure.

## References

[1] CW Ma R CN Chan J. Type 2 diabetes in East Asians: similarities and differences with populations in Europe and the United States. Ann N Y Acad Sci (2013) 1281:64–91. 10.1111/nyas.12098 23551121 PMC3708105

[2] Cho NH Shaw JE Karuranga S Huang Y da Rocha Fernandes JD Ohlrogge AW. Idf diabetes atlas: global estimates of diabetes prevalence for 2017 and projections for 2045. Diabetes Res Clin Pract (2018) 138:271– 281. 10.1016/j.diabres.2018.02.023 29496507

[3] Lin CH Chang DM Wu DJ Peng HY Chuang LM. Assessment of Blood Glucose Regulation and Safety of Resistant Starch Formula-Based Diet in Healthy Normal and Subjects With Type 2 Diabetes. Medicine (2015) 94:(33): e1332. 10.1097/MD.0000000000001332 26287417 PMC4616456

[4] Murea M Ma L Freedman BI. Genetic and environmental factors associated with type 2 diabetes and diabetes vascular complications. Rev Diabetes Stud (2012) 9(1):6–22. 10.1900/RDS.2012.9.6 PMC3448170 22972441

[5] Williams SB Cusco JA Roddy MA Johnstone MT Creager MA. Impaired nitric oxide-mediated vasodilation in patients with non-insulin-dependent diabetes mellitus. J Am Coll Cardiol (1996) 27(3):567–74. 10.1016/0735-1097(95)00522-6 8606266

[6] Senoner T Dichtl W. Oxidative Stress in Cardiovascular Diseases: Still a Therapeutic Target? Nutrients (2019) 11(9):pii: E2090. 10.3390/nu11092090 PMC6769522 31487802

[7] Wang S Li J Zhang C Xu G Tang Z Zhang Z. Effects of aerobic exercise on the expressions and activities of nitric oxide synthases in the blood vessel endothelium in prediabetes mellitus. Exp Ther Med (2019) 17(5):4205–12. 10.3892/etm.2019.7437 PMC6468937 31007752

[8] Odegaard AO Jacobs DR Jr Sanchez OA Goff DC Jr Reiner AP Gross MD. Oxidative stress, inflammation, endothelial dysfunction and incidence of type 2 diabetes. Cardiovasc Diabetol (2016) 15:51. 10.1186/s12933-016-0369-6 27013319 PMC4806507

[9] Kodolova IM Lysenko IV Saltykov BB. Change in the lung in diabetes mellitus. Arkh Pathol (1982) 44(7):35–40. 7125937

[10] Kwon CH Rhee EJ Song JU Kim JT Kwag HJ Sung KC. Reduced lung function is independently associated with increased risk of type 2 diabetes in Korean men. Cardiovasc Diabetol (2012) 11:38. 10.1186/1475-2840-11-38 22524685 PMC3464774

[11] American Diabetes Association. “Standards of medical care in diabetes 2007”. In: Diabetes Care, vol. 30. (2007). p. S4–S41. 10.2337/dc07-S004 17192377

[12] Hahad O Wild PS Prochaska JH Schulz A Hermanns I Lackner KJ. Endothelial Function Assessed by Digital Volume Plethysmography Predicts the Development and Progression of Type 2 Diabetes Mellitus. J Am Heart Assoc (2019) 8(20):e012509. 10.1161/JAHA.119.012509 31583936 PMC6818038

[13] Bondonno CP Yang X Croft KD Considine MJ Ward NC Rich L. Flavonoid−rich apples and nitrate−rich spinach augment nitric oxide status and improve endothelial function in healthy men and women: A randomized controlled trial. Free Radic Biol Med (2012) 52(1):95−102. 10.1016/j.freeradbiomed.2011.09.028 22019438

[14] Tatsch E Bochi GV Pereira Rda S Kober H Agertt VA de Campos MM. A simple and inexpensive automated technique for measurement of serum nitrite/nitrate. Clin Biochem (2011) 44(4):348–50. 10.1016/j.clinbiochem.2010.12.011 21185277

[15] Meigs JB O’Donnell CJ GH Tofler EJ B CS F Lipinska I. Hemostatic markers of endothelial dysfunction and risk of incident type 2 diabetes: the Framingham Offspring Study. Diabetes (2006) 55(2):530–7. 10.2337/diabetes.55.02.06.db05-1041 16443791

[16] Rossi R Cioni E Nuzzo A Origliani G Modena MG. Endothelial-dependent vasodilation and incidence of type 2 diabetes in a population of healthy postmenopausal women. Diabetes Care (2005) 28(3):702–7. 10.2337/diacare.28.3.702 15735211

[17] Tousoulis D Kampoli AM Stefanadis C. Diabetes mellitus and vascular endothelial dysfunction: current perspectives. Curr Vasc Pharmacol (2012) 10(1):19–32. 10.2174/157016112798829797 22112354

[18] Flammer AJ Anderson T Celermajer DS Creager MA Deanﬁeld J Ganz P. The assessment of endothelial function: from research into clinical practice. Circulation (2012) 126(6):753–67. 10.1161/CIRCULATIONAHA.112.093245 PMC3427943 22869857

[19] Rubinshtein R Kuvin JT Soffler M RJ L Lavi S RE N. Assessment of endothelial function by non-invasive peripheral arterial tonometry predicts late cardiovascular adverse events. Eur Heart J (2010) 31(9):1142–8. 10.1093/eurheartj/ehq010 20181680

[20] Samsamshariat SZA Sakhaei F Salehizadeh L Keshvari M Asgary S. Relationship between Resistin, Endothelin-1, and Flow-Mediated Dilation in Patient with and without Metabolic Syndrome. Adv BioMed Res (2019) 27(8):16. 10.4103/abr.abr_126_18 PMC6425749 30993086

[21] Weil BR Westby CM Van Guilder GP Greiner JJ Stauffer BL DeSouza CA. Enhanced endothelin-1 system activity with overweight and obesity. Am J Physiol Heart Circ Physiol (2011) 301(3):H689−695. 10.1152/ajpheart.00206.2011 21666117 PMC3191085

[22] Chen C Jiang J Lü JM Chai H Wang X Lin PH. Resistin decreases expression of endothelial nitric oxide synthase through oxidative stress in human coronary artery endothelial cells. Am J Physiol Heart Circ Physiol (2010) 299(1):H193−201. 10.1152/ajpheart.00431.2009 20435848 PMC2904138

[23] Lukashevich V Del Prato S Araga M Kothny W. Efficacy and safety of vildagliptin in patients with type 2 diabetes mellitus inadequately controlled with dual combination of metformin and sulphonylurea. Diabetes Obes Metab (2014) 16(5):403–9. 10.1111/dom.12229 PMC4237555 24199686

[24] Prabhu M Kakhandaki A Chandra KR Dinesh MB. A Hospital Based Study Regarding Awareness of Association Between Glycosylated Haemoglobin and Severity of Diabetes Retinopathy in Type 2 Diabetes Individuals. J Clin Diagn Res (2016) 10(1):NC01–4. 10.7860/JCDR/2016/15834.7014 PMC4740628 26894100

[25] Cavan DA Parkes A O’Donnell MJ Freeman W Cayton RM. Lung function and diabetes. Respir Med (1991) 85(3):257–8. 10.1016/s0954-6111(06)80092-2 1882118

[26] Hsia CC Raskin P. Lung function changes related to diabetes mellitus. Diabetes Technol Ther (2007) 9(Suppl 1):S73–82. 10.1089/dia.2007.0227 17563307

[27] Barnes PJ. The role of inflammation and anti-inflammatory medication in asthma. Respir Med (2002) 96(Suppl. A):S9–15. 11858564

[28] Niranjan V McBrayer DG Ramirez LC Raskin P Hsia CC. Glycemic control and cardiopulmonary function in patients with insulin-dependent diabetes mellitus. Am J Med (1997) 103(6):504–13. 10.1016/s0002-9343(97)00251-9 9428834

[29] Davis TM Knuiman M Kendall P Vu H Davis WA. Reduced pulmonary function and its associations in type 2 diabetes: the Fremantle Diabetes Study. Diabetes Res Clin Pract (2000) 50(2):153–9. 10.1016/s0168-8227(00)00166-2 10960726

[30] Lange P Groth S Kastrup J Mortensen J Appleyard M Nyboe J. Diabetes mellitus, plasma glucose and lung function in a cross-sectional population study. Eur Respir J (1989) 2(1):14–9. 10.1007/s11154-013-9239-7 2651148

[31] Miller JA. Impact of hyperglycemia on the renin angiotensin system in early human type 1 diabetes mellitus. J Am Soc Nephrol (1999) 10(8):1778–85. 10.1038/nature11464 10446946

[32] Muris DM Houben AJ Schram MT Stehouwer CD. Microvascular dysfunction is associated with a higher incidence of type 2 diabetes mellitus: a systematic review and meta-analysis. Arterioscler Thromb Vasc Biol (2012) 32(12):3082–94. 10.1161/ATVBAHA.112.300291 23042819

[33] Eringa EC Serne EH Meijer RI Schalkwijk CG Houben AJ Stehouwer C. Endothelial dysfunction in (pre) diabetes: characteristics, causative mechanisms and pathogenic role in type 2 diabetes. Rev Endocr Metab Disord (2013) 14(1):39–48. 10.1007/s11154-013-9239-7 23417760

[34] Hagberg CE Mehlem A Falkevall A Muhl L Fam BC Ortsater H. Targeting vegf-b as a novel treatment for insulin resistance and type 2 diabetes. Nature (2012) 490(7420):426–30. 10.1038/nature11464 23023133

[35] Giroix MH Irminger JC Lacraz G Noll C Calderari S Ehses JA. Hypercholesterolaemia, signs of islet microangiopathy and altered angiogenesis precede onset of type 2 diabetes in the goto-kakizaki (gk) rat. Diabetologia (2011) 54(9):2451–62. 10.1007/s00125-011-2223-4 21744291

[36] Tai H Wang MY Zhao YP Li LB Jiang XL Dong Z. Pulmonary Function and Retrobulbar Hemodynamics in Subjects With Type 2 Diabetes Mellitus. Respir Care (2017) 62(5):602–14. 10.4187/respcare.05129 28246307

